# Predicting species diversity and community traits from remote sensing in species-rich grasslands

**DOI:** 10.1186/s12862-026-02500-4

**Published:** 2026-02-13

**Authors:** Samantha Suter, Natalie Welden, Kenny Roberts, Brian Barrett

**Affiliations:** 1 School of Social and Environmental Sustainability, University of Glasgow, Glasgow, UK; 2 School of Geographical and Earth Sciences, University of Glasgow, Glasgow, UK; 3 Institute of Biology, University of Neuchâtel, Neuchâtel, Switzerland

**Keywords:** Remote sensing, Species-rich grasslands, Grassland monitoring, Spectral variation hypothesis, Biodiversity, Community traits, Grassland trait prediction

## Abstract

Species-rich grasslands (SRGs) provide unique ecosystem services, yet they are some of the most understudied environments in Scotland. This is because their true extent is unknown, and their locations are often in unreachable areas. Remote sensing may ameliorate this by helping to distinguish between SRG classes and increase monitoring efforts. However, these applications have not been conducted across highly diverse grasslands at national scales. We aimed to address this by applying remote sensing techniques to multiple classes of species-rich grasslands, nationally. Using an Unmanned Aerial Vehicle (UAV), we investigated the Spectral Variation Hypothesis, testing the relationship between species diversity (species richness) and spectral diversity (variation in surface reflectance using the coefficient of variation and standard deviation). Additionally, data was acquired from Sentinel-2 and Planetscope satellites, as well as the UAV data, assessing the prediction of grassland community traits (above ground biomass, sward height, and SPAD-measured chlorophyll) across multiple spatial (8 cm − 10 m) and spectral (8–13 bands) resolutions. The results contrast with prior studies of single sites and less diverse grasslands, indicating that there was no significant relationship between species diversity and the spectral diversity metrics, standard deviation (*p* = 0.120) and the coefficient of variation (*p* = 0.141), across seven of our study sites. The accuracy of grassland trait prediction varied largely with spatial and spectral resolution and the combination of predictor variables used. The UAV mounted multispectral Micasense predictor variables were most successful in predicting all traits: sward height, above ground biomass, and SPAD-measured chlorophyll (R^2^ = 0.545, R^2^ = 0.221, R^2^ = 0.167 respectively). The results suggest monitoring across species-rich grassland classes using remote sensing may be hampered by increased variation and confounding factors in these highly diverse habitats. Further methodological advancements are needed for wide scale cross-grassland habitat differentiation and monitoring, and field guidelines for remote sensing species-rich grasslands must be elaborated.

**Supplementary Information:**

The online version contains supplementary material available at 10.1186/s12862-026-02500-4.

## Introduction

Grassland ecosystems provide a range of benefits, such as carbon sequestration, forage production, habitat for vulnerable species, nutrient recycling, and flood mitigation [1], yet almost 50% of the world’s grasslands are considered degraded [2]. This degradation is largely associated with land-use change, management intensification or land abandonment, and climate change [3]. Species-rich grasslands (SRGs) – defined by their high floristic diversity of > 12–15 species per square meter, with a herbaceous cover greater than 30%, low productivity, and not under agricultural use – are amongst the most impacted [4]. For example, in the UK, only 3% of initial SRGs remain intact [5]. Because of the vital ecosystem services that grasslands provide, long-term monitoring and restoration of grassland ecosystems have been identified as key conservation goals [6]. To reach these goals, surveys detailing the extent and quality of the habitat of these sites are needed first, followed by accurate mapping of their locations (as outlined by the EU’s biodiversity Strategy) [7–9].

Typically, we see mapped grassland ecosystems only discriminated between agricultural grasslands (otherwise called improved) and semi-natural grasslands. The former consists of grassland that is agriculturally enhanced through the addition of chemicals and heavy grazing (typically sheep or cattle) and/or frequent cutting or mowing. The latter, although a human modified habitat, differs through minor grazing input from wild or domestic herbivores or infrequent mowing [10]. SRGs themselves belong to a small subset of the wider classification of semi-natural grasslands. However, various classes of SRGs exist, historically differentiated into acidic, neutral, and calcareous (and marshy) classes [11]. Each SRG type provides unique habitat to an array of species and contribute differently to the ecosystem services, and, as such, require individual targeted monitoring [12]. However, the varying phenological, functional, and species diversity (collectively community) traits of these SRG classes are often difficult to separate. The gradient from agricultural to more semi-natural grasslands was described by Sullivan et al. [13] over a decade ago, demonstrating how difficult the differentiation between grassland classes can be.

Evidence shows that grassland trait retrieval is possible by optical remote sensing (RS), through modelling the relationship between traits and spectral responses (e.g. [14, 15]). Studies in grassland monitoring have demonstrated the value of remote sensing, taking advantage of increased spatial and spectral resolution of airborne and spaceborne sensors (e.g. [16–19]). Successfully retrieved traits include biochemical properties (such as water content, chlorophyll, and nutrient composition), and biophysical properties (such as leaf area or biomass) [20–22], often retrieved from hyperspectral sensors such as Hyperion [23]. By estimating chlorophyll content, we may differentiate SRGs from improved grasslands through varying fertiliser application, for example [24, 25].

Furthermore, plant functional and structural traits distinguish community and grassland productivity – a key factor in differing agricultural grasslands from semi-natural grasslands, such as SRGs [26]. For instance, sward height and above ground biomass (AGB) can often indicate management practices such as grazing/cutting frequency. In fact, AGB estimation has one of the largest applications in RS of grasslands [19] which has often been achieved with high accuracy from multiple sensors including Landsat, MODIS, and Sentinel-2 [27, 28]. On the other hand, LiDAR is often used to estimate other structural traits such as sward height [29]. Deriving vegetation indices from RS may further aid in monitoring of grasslands, most typically the Normalised Difference Vegetation Index (NDVI) [23], which assesses the level of greenness and is also indicative of potential biomass.

There have been previous issues of monitoring these landscapes with RS due the existence of grassland features at a fine-spatial scale, especially those that are heterogenous and diverse [23, 30]. Nuanced discrimination between intra-grassland classes is currently not wide scale, despite existing academic interest [31, 32]). In fact, grassland monitoring from RS typically focus on productivity and forage quality assessment, for example, more applicable in agricultural landscapes [27, 33]. However, studies using RS to assess more natural grasslands are growing, especially with the use of UAV sensors [34]. One such application of biodiversity RS is that of the Spectral Variation Hypothesis. The Spectral Variation Hypothesis theorises that increased species diversity leads to increased spectral diversity [35]. This has been demonstrated in a range of grassland conditions, such as monocultures [36], farmland [37], and prairie [38] and spectral diversity seems particularly plausible in determining alpha (within community) and beta (between community) diversity [39, 40]. 

However, variability between studies is found due to the complexity across and within systems [41]. Issues with the hypothesis are seen at landscape level, where landcover has affected the relationship between species and spectral diversity [42], as have the confounding effect of spatial resolution, for example, which influence the spectral response [30, 43].

Previously, RS of grasslands has largely used MODIS or Landsat satellites, but research has shown that increased spatial, spectral, and temporal resolution are needed for improved grassland observation [16, 30, 44–47]. The use of the newer Sentinel-2 satellites has had more attention due to their higher spatial resolution (10–60 m), spectral resolution (13 bands), revisit frequency (5 days), and free access, particularly in relation to assessment of functional, spectral, and species diversity as well as productivity in grasslands [48–50]. We now see greater interest in the use of Sentinel-2, which has experienced the largest growth in grassland applications since its launch in 2015 [51]. Furthermore, with the Open Science movement growing [52], open-source satellite imagery should be at the forefront of applications.

Additionally, image fusion and data combinations with higher spatial resolution satellites, such as PlanetScope (3 m resolution) or Unmanned Aerial Vehicles (UAV) (cm level resolution) have been shown to improve accuracy in grassland RS applications [16, 53]). Combining multiple datasets may provide more robust methods of grassland observation, rendering it necessary to investigate the use of various RS sensors in applications of grassland monitoring. With most studies focusing on less diverse grasslands, greater exploration of RS applications in heterogenous grasslands is needed.

To assess if RS can improve widescale grassland monitoring and help distinguish between the multiple classifications of SRG types, this study aimed to investigate the relationship between species diversity, spectral diversity, and the retrieval success of grassland community traits in SRGs found across Scotland. These applications are seldom executed in biodiverse grasslands, especially across multiple intra-grassland classes of SRGs at a national scale. This is the first instance of such a study on SRGs of Scotland.

Scotland has little data coverage on the extent of this priority habitat. Estimates suggest that only 2.6% of undesignated SRG sites, of approximately 30,000 ha found in Scotland, are known [54]. Existing records of SRGs in Scotland are dated to the end of the 20^th^/early 21^st^ century. As of 2019, National Vegetation Classification surveys (that classify plant communities) have only been conducted for 15% of Scotland, with more than 60% of these occurring before 2010 [55]. This is problematic as these habitats have likely undergone land use changes since assessment – Mitchell et al. [56] found that overall diversity in Scottish grasslands was decreasing, with some species becoming dominant because of changing management, leading to an extinction debt. Moreover, difficult topography and areas of inaccessibility make comprehensive mapping and monitoring of this habitat problematic with traditional techniques [57]. Therefore, RS may be a viable means to aid in the mapping and monitoring of this habitat, by predicting areas of higher species richness and retrieving traits that may differentiate between similar classes of SRGs

As such, our specific objectives included: i) investigating the relationship between species and spectral diversity across seven SRG sites; and ii) estimating plant community structural and biochemical traits, such as Soil Plant Analysis Development (SPAD)- measured proxy for chlorophyll, sward height, and AGB using various RS sensors across spatial and spectral scales, in eleven SRG sites.

## Methods

### Study sites

**Fig. 1:**
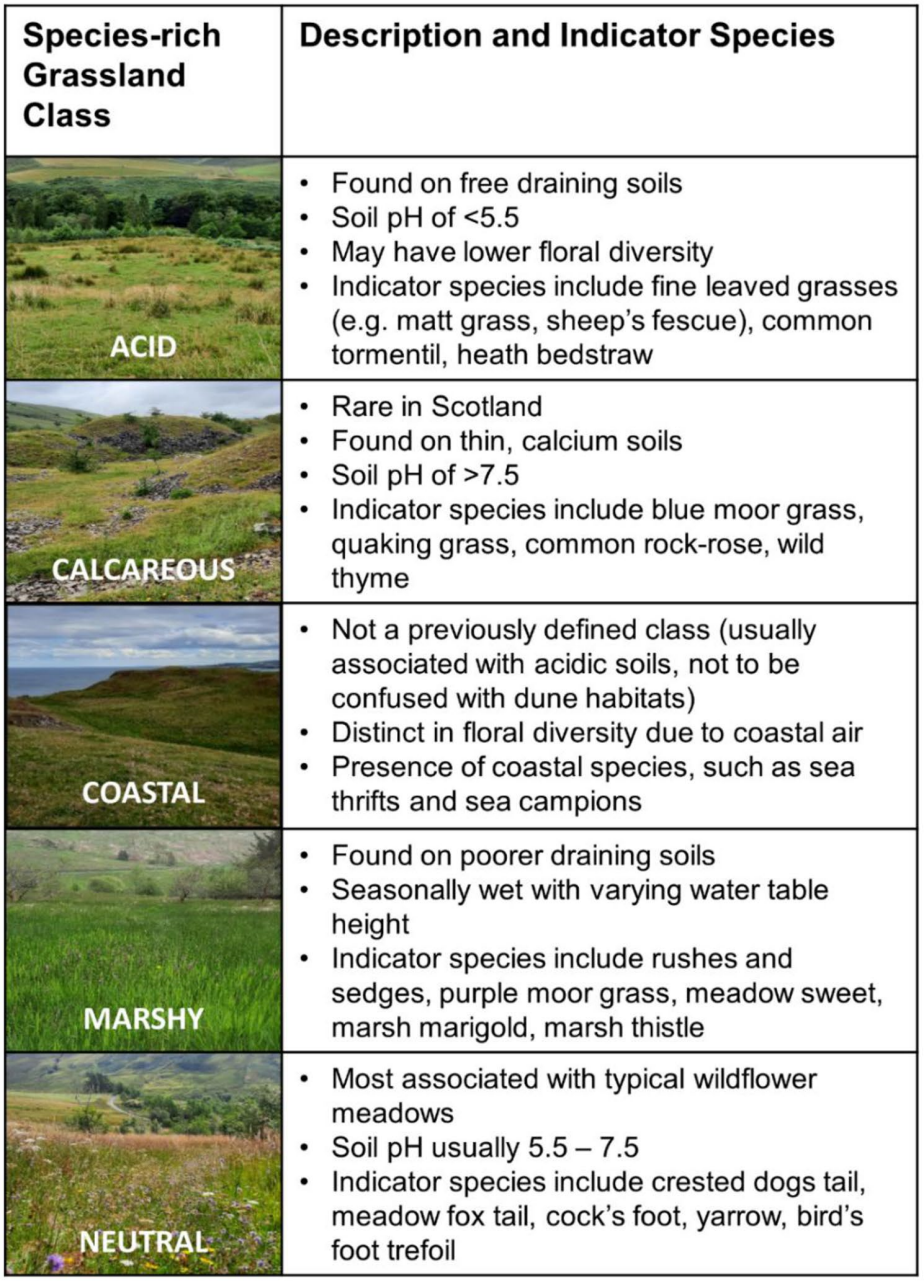
Five types of species-rich grasslands in Scotland analysed in this study

Over the grass growing season (May – August) in 2022, surveys were undertaken at eleven sites of SRGs across Scotland (56.49° N, 4.20° W). Scotland forms the northern part of the United Kingdom, displaying distinct geology and high topographical variation reaching up to 1345 m above sea level [58]. Sitting in the temperate zone, the country experiences a large range in climatic conditions with annual precipitation varying between 600 and 3000 m and mean annual temperatures ranging between (4–11 °C) [59]. As such, multiple habitat types exist, including a range of semi-natural SRGs. The eleven sites of SRGs visited are made up of five typical SRG classes found in Scotland, acid, neutral, calcareous, marshy, and coastal, ranging in abiotic and biotic conditions (Fig. 1).

**Fig. 2:**
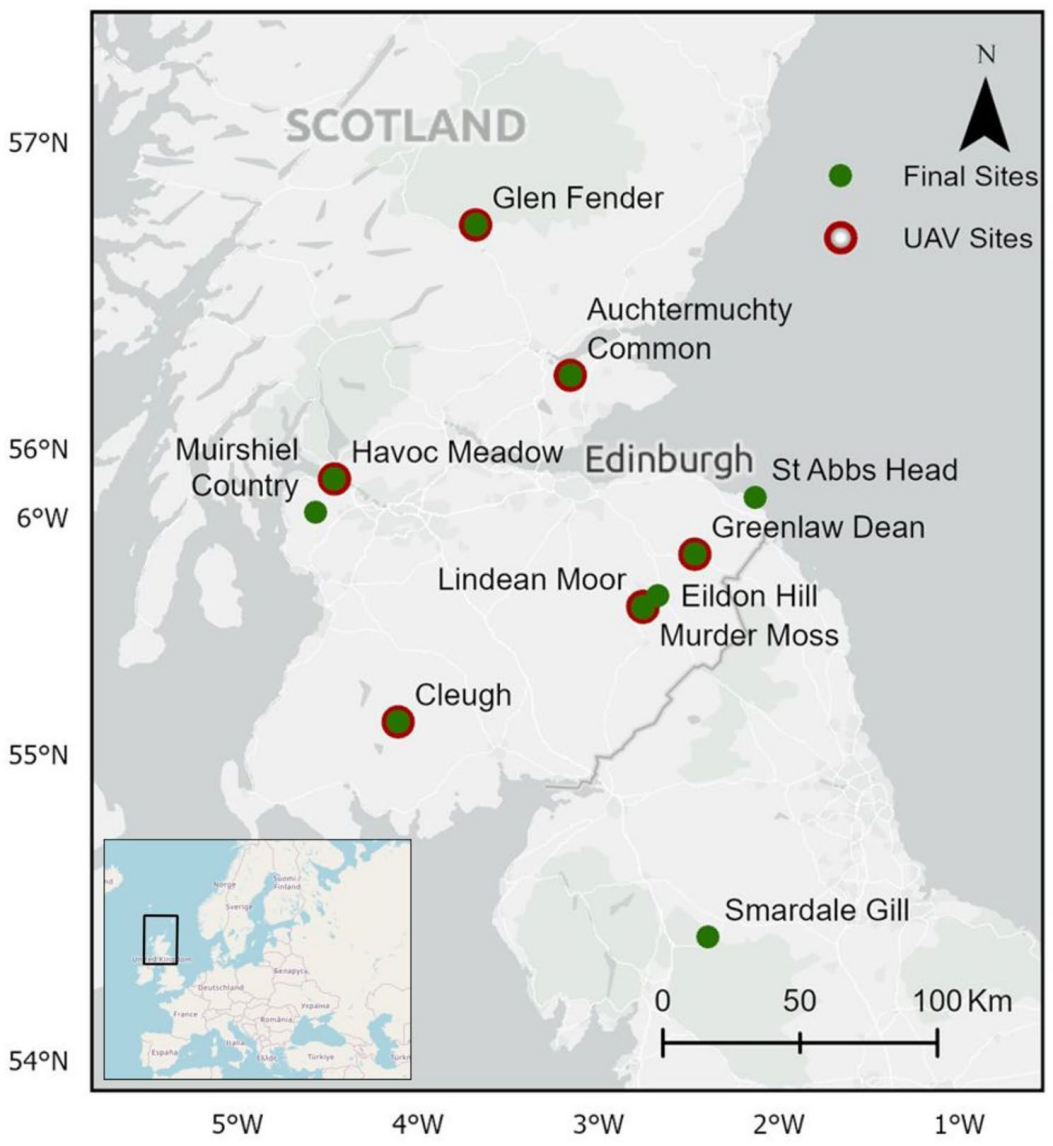
Species-rich grassland sites located across Scotland sampled in this study in 2022. UAV data was acquired at seven of the eleven sites (highlighted in red). Lindean Moor and Murder Moss are represented as one location point due to proximity. Scotland’s location in Europe is highlighted in the insert (bottom left)

Open-source data (from Butterfly Conservation, NatureScot, and the UK Centre of Ecology and Hydrology) on previous and potential SRG locations were used to identify sites. The final eleven sites were chosen based on accessibility and to ensure a representation of the five SRG classes (as described above) (Fig. 2).

The sites differed in their geology, topography, and climates, as they are located across Scotland and, as such, provide a representation of the SRGs that can be found across the country. Site management also varied: seven were lightly grazed, two included cutting regimes (cut one year and grazed/left the next), two were unmanaged, and one had the use of herbicide application. One site was accidentally trampled in the late summer of 2022.

### In situ data collection and lab processing

**Fig. 3:**
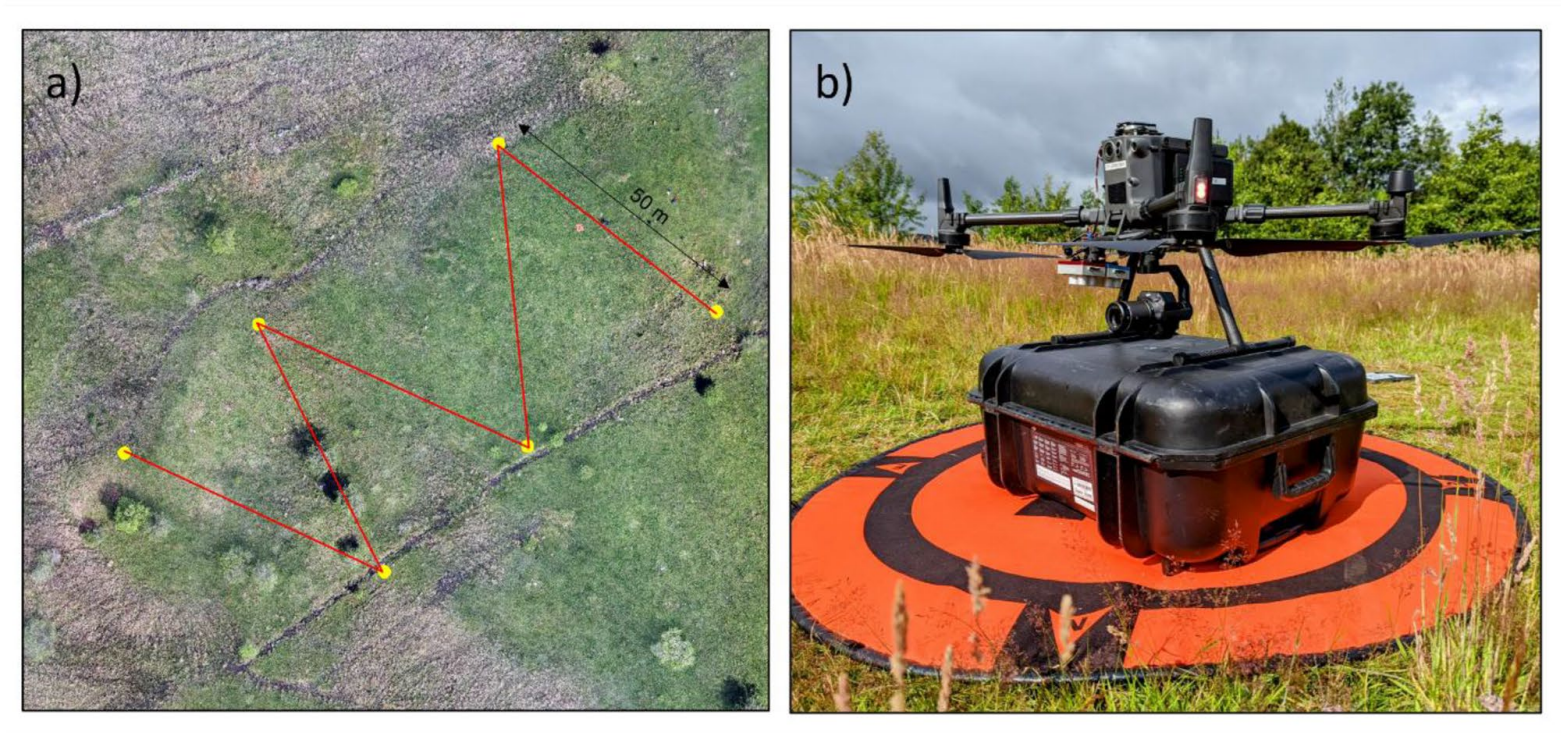
**a**) Orthophoto of a site (Lindean moor, see Fig. 2) detailing the “W” transect methodology employed to survey each site. The “W” transects were made up of 5 × 50 m (total = 250 m) splits, each ending with a 0.5 × 0.5 m quadrat; **b**) DJI matrice 300 RTK UAV platform that was used for all acquisitions with a Micasense Rededge MX 5.5 dual camera and DJI zenmuse P1 sensor

Each of the eleven sites was visited three times over the summer growing season in the same year. A fixed 250 m “W” transect (the origin of which was selected randomly by throwing a quadrat, see. Fig. 3a) was used to gain representative community trait variation over the habitat [60, 61].

The transect length was constrained by varying site sizes (found between < 1–326 ha) and surveyable areas (i.e., areas devoid of trees, shrub, site boundaries, and water features), and the 250 m length was chosen to ensure consistency between sites. A total of six 0.5 × 0.5 m quadrats were used along the transect at 50 m intervals to determine community traits and species richness. These were georeferenced with a Garmin eTrex 10, with an accuracy of 3 m. Recorded variables were selected based on the community traits (AGB, sward height, and SPAD-measurements as a proxy for chlorophyll) that vary between SRG classes. In each quadrat, species richness was determined by counting the total number of species [30]. Species richness was used as the species diversity metric in the analysis. Sward height was measured randomly five times within a quadrat using a tape measure and the tallest part of the vegetation (avoiding inflorescences) that touched the tape was recorded in each quadrat and then averaged. Subsequently, the ABG was removed by clipping to grazing level in a quadrat.

On return to the laboratory, this material was oven dried (oven LTE OP250 40 °C to 250°C) at 70 °C until a constant weight was reached. The dry weight was then calculated per area unit. Chlorophyll content could not be measured with a spectrophotometer due to equipment and time limitations. As such, a chlorophyll proxy was used, measured in situ with a SPAD meter (Konica Minolta SPAD-502 Plus) on five randomly chosen leaves across the quadrat. The SPAD meter was calibrated before each use. The five values were then averaged to give a SPAD value per quadrat.

### Satellite and UAV acquisitions and processing

Satellite data was captured across all eleven sites including neutral, calcareous, acid, and coastal SRGs, whereas it was only possible to acquire UAV data across seven of the eleven sites, all consisting of neutral SRGs: limited by access, permissions, and weather (Table 1).

**Table 1:** The dates of the field surveys for the eleven SRG sites surveyed in this study in 2022. Dates highlighted in bold indicate when unmanned aerial vehicle surveys occurred in conjunction with the field surveys. The corresponding dates of satellite acquisitions of Sentinel-2 (S2) and Planetscope (PS) imagery that are associated with the field survey dates are listed. *list of site names, corresponding codes, geographic coordinates, and site elevation can be found in* Table S1

Site	May			June/July			August		
	Field Survey	S2	PS	Field Survey	S2	PS	Field Survey	S2	PS
AC	**24/05**	19/05	08/05	**12/07**	18/07	07/07	**16/08**	10/08	11/08
CL	**19/05**	27/05	26/05	**30/06**	04/07	10/07	**04/08**	10/08	04/08
EH	17/05	24/05	27/05	28/06	18/07	07/07	02/08	10/08	09/08
GD	**17/05**	14/05	22/06	**28/06**	18/07	07/07	**02/08**	10/08	10/08
GF	25/05	25/04	05/06	**13/07**	09/07	10/07	**17/08**	10/08	17/08
HM	26/05	04/06	29/05	**14/07**	09/07	10/07	**18/08**	10/08	11/08
LM	**16/05**	24/05	08/05	**27/06**	18/07	22/06	**01/08**	10/08	09/08
MM	**16/05**	24/05	08/05	**27/06**	18/07	22/06	**01/08**	10/08	09/08
MP	26/05	04/06	29/05	14/07	09/07	18/07	18/08	20/08	12/08
SA	23/05	24/05	24/05	11/07	18/07	10/07	15/08	10/08	10/08
SM	18/05	14/05	18/05	05/07	16/07	09/07	03/08	10/08	10/08

We used sun-synchronous Sentinel-2A and −2B (orbital altitude 786 m; orbital inclination 98.62°) and Planetscope (orbital altitude 475–525 km; orbital inclination 98°) satellites. Sentinel-2 (S2) data was acquired due to its high spectral resolution of 13 bands, medium spatial resolution of 10–60 m (Table S2), and high revisitation frequency (5 days), as well as being open-source data. Level-2 atmospherically corrected surface reflectance imagery was acquired using Google Earth Engine for each site, as close as possible to survey dates (±0–31 days, see Table 1 for corresponding dates). Specific S2 bands (B2, B3, B4, B8 at 10 m resolution and B5, B6, B7, B8A, B11, B12 at 20 m resolution) associated with vegetation characteristics were selected for use in image classification and resampled (nearest neighbour) to the highest 10 m resolution.

Planetscope (PS) surface reflectance data were also acquired due to its higher spatial resolution of 3 m and revisit frequency of one day, however, it has a reduced spectral resolution of 8 bands (Table S2). This allowed comparison between spectral and spatial resolution limitations, between commercial and open data sources. The Raster package [62] in R (v 3.6.3) was used to extract the satellites’ reflectance values for each pixel corresponding to the quadrat location.

At the seven neutral SRG sites, a DJI Matrice 300 RTK UAV platform (see Fig. 3b) was used and flown at 50 m, with an 80% front and side overlap. We used 7–10 ground control points on each site for accurate positioning, using a Leica Viva GS08 GNSS receiver. A Micasense (MS) Red-edge MX 5.5 dual camera was used to acquire hyperspatial (8 cm) and multispectral (10 band) images (Table S2). This sensor allows bands to be synergised with S2 bands, as well as providing hyperspatial resolution of multispectral data. The reflectance was calibrated in field using a white reflectance panel (RP06-2102083-OB).

Subsequently, Pix4D Mapper (v 4.6.4) was used to create orthomosaics and surface reflectance images of the sites (georeferenced between 0.009–0.054 m), and 0.5 × 0.5 m polygons were created over the quadrats in ArcGIS Pro (v 3.0.36057). The R Raster package [62] was then used to extract the reflectance values of each pixel within a quadrat, creating an average across the polygon areas for each of the 10 MS bands.

Vegetation indices (VIs) were calculated from the spectral reflectance values, as the ratio between bands is often more sensitive to variables on the ground (as seen in the positive relationship between the Normalised Difference Vegetation Index (NDVI) and AGB) [63, 64]. As such, several VIs were calculated: the NDVI which often discriminates between land use types and for biomass estimation [63, 64] the red-edge NDVIs and the Sentinel-2 Red-edge position index, S2REP, which represent information on chlorophyll content [65, 66]; the enhanced vegetation index (EVI), used in association with species diversity estimates [67]; the greenness index (GVI), which may help discriminate between grassland classes [68], and the normalised difference infrared index (NDII) sensitive to water changes in plants [66] (see Table S3).

### Statistical analysis

Overall, 198 quadrats were surveyed across sites. However, due to missing information from three quadrats, the total dataset comprises 195 quadrats. For the analysis associated with the spectral variation hypothesis, the dataset is restricted to 114 quadrats across seven sites, where the UAV acquisitions were possible.

#### Species – spectral diversity relationship

The MS UAV data (*n = 114*) was used to evaluate the relationship between spectral and species diversity. Spectral diversity metrics included were the standard deviation (SD) and coefficient of variation (CV) (Eq. 1), often shown to have the most predictive power in diversity estimations [38, 67, 69]. Other metrics, such as Convex Hull Volume, are more easily influenced by outliers [70]. Clustering methods were not used; Rao’s Q (calculated as the distance between the reflectance of two random pixels) is more applicable for assessing functional (abundance weighted) diversity which could not be measured in this study, whilst the Spectral Species approach is less suitable for cross-site studies [30, 71, 72].

Both SD and CV spectral metrics were calculated by averaging individual SD and CV values across the pixels in a quadrat (0.5 ×0.5 m). For this reason, only MS data was applicable for this objective, as S2 and PS pixel areas exceeded the quadrat area (10 ×10 m and 3 × 3 m versus 0.5 × 0.5 m). Due to the coarser scale of the satellites’ pixels compared to the quadrat, only one pixel of S2 or PS covers the quadrat area (unlike the multipixel coverage from the MS). As such, no variation in spectral reflectance (i.e. no spectral diversity) can be calculated from the satellite sensors for this analysis. Extrapolating the satellite data over an increased area was not applicable for these sites due to the mosaiced habitats.

An NDVI soil mask (pixel values ≥ 0.4) and a NIR shade mask (pixel values ≥ 0.22) were applied to the UAV data [73], however, it was observed that most quadrats were fully vegetated ( > 99% of pixels), thus soil was unlikely to be an influencing factor. Due to the structure and composition of the grassland species, and the avoidance of trees/shrub close to quadrats, shadows were unlikely to confound the reflectance values ( > 99% of pixels above threshold). The average SD and CV of spectral reflectance values across the visible and near infrared wavelengths (444–842 nm) were used. CV was calculated as the standard deviation/mean reflectance value at a specific band, then averaged across all 10 bands of the MS, for each quadrat (as in Eq. 1).

1 $$C{V_{quadrat}} = {\rm{ }}\sum {} \left( {S{D_{wavelength}}/Mea{n_{wavelength}}} \right)/number{\rm{ }}of{\rm{ }}bands$$

The relationships between spectral – species diversity was tested using a Pearson Correlation coefficient (r). After visualising and analysing the distribution of both metrics, Spectral Diversity SD was logarithmic (log) transformed to ensure the test assumptions were met. The predictive power of the spectral diversity metrics SD and CV on species diversity was then estimated with the coefficient of determination (R^2^), resulting from the Pearson’s Correlation.

#### Species-rich grassland community trait retrieval

Both satellite (S2 and PS) and UAV (MS) data were used to investigate grassland community trait estimation from RS data, to allow for a spatial and spectral resolution comparison, where we took the pixel from each sensor that overlapped with the location of the quadrat. The partial least square regression model (PLS) was chosen to evaluate RS data in predicting grassland community structural (AGB and sward height) and functional (SPAD) traits. PLS is a non-parametric model which works well with highly colinear data, such as environmental data, and is commonly used for trait estimation from spectral data due to its consistent ability to outperform other models in prediction analyses [74–77].

**Fig. 4:**
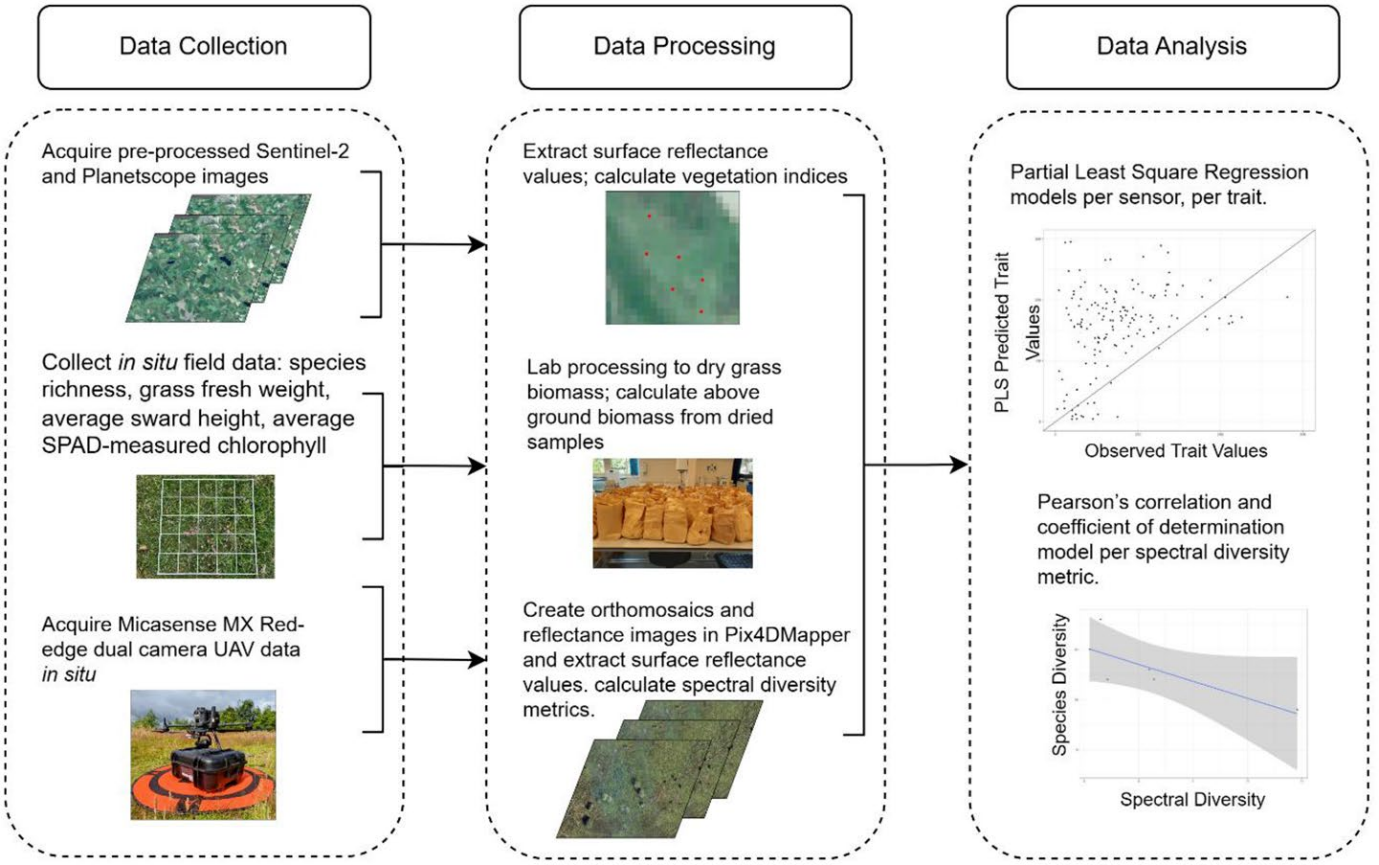
Processing steps involved in model creation for predicting species richness and grassland community traits in species-rich grasslands across Scotland

Both surface reflectance data of relevant bands in vegetation analysis (spectral range) and VIs were used as predictor variables, as seen in previous studies [22, 74, 75]. The spectral diversity metrics of the MS were also included in the PLS MS models to explore its potential influence on trait predictions. Further information on this analysis can be found in the Supplementary Materials 1. PLS models were created for S2 data (PLS-S2), PS data (PLS-PS), and for MS data (PLS-MS). For each dependent variable, the model was originally created with all predictor components. Each sensor had differences in the number of bands, VIs, and whether there were spectral metrics or not. As such, multiple combinations of the predictor components were tested in each model to investigate the most relevant bands and VIs for the highest prediction accuracy per trait estimation. For example, model iterations included the sole removal of NIR bands, the sole removal of RGB bands, the sole removal of SWIR bands, and the sole removal of edge bands (such as the red-edge bands). This is because different regions of the spectrum hold varying information on plant properties depending on the interaction at particular wavelengths [78]. For example, high plant water content would result in high absorption in the SWIR bands; greater chlorophyll content is absorbed more strongly in Red-Edge wavelengths, peaking in the NIR region [79]. Thus, these iterations could remove noise introduced from other areas of the spectrum for improved trait retrieval accuracy. All models were trained and tested with a 70:30% split. A 10-fold cross validation was performed on each model and the best tuned model was chosen as the final model per trait prediction based on the highest R^2^ values. The data collection and analysis workflow can be visualised in Fig. 4.

## Results

### Site descriptive statistics

**Fig. 5:**
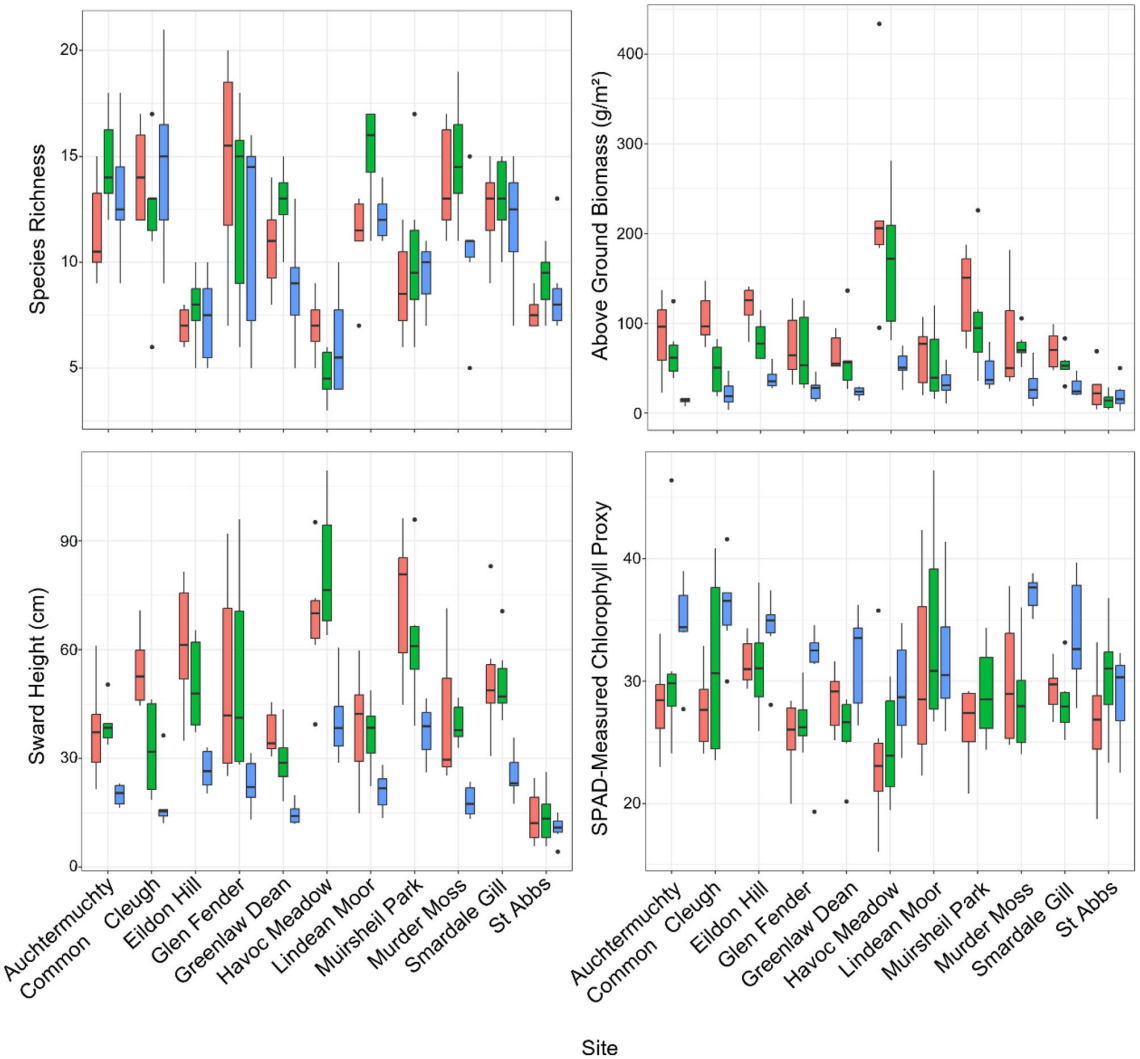
Variation in grassland community traits (species richness, above ground biomass (g/m^2^), average sward height (cm), average Soil Plant Analysis Development (SPAD) used as a proxy for chlorophyll, across 11 species-rich grassland sites in Scotland over the grass-growing season: May (pink), June/July (green), and August (blue)

SRG species numbers and community trait values varied largely across the SRG sites due to the heterogeneity seen both within and between classes (Fig. 5). Species richness was lowest in Havoc Meadow, with an average of six species per quadrat, whereas the highest species richness was found at Cleugh, with an average of 14 species per quadrat. Species richness had a large range from 3 to 21 species found in a quadrat, however, across the sites. Average sward height of a quadrat ranged from 4.24 cm at St Abb’s to 109.38 cm at Havoc Meadow, with the average sward height peaking (81.96 cm) in June at Havoc Meadow. Similarly, St Abb’s had the lowest recorded quadrat AGB of 2.16 g/m^2^, whilst the highest quadrat AGB recorded was at Havoc Meadow of 433.6 g/m^2^. The mean quadrat AGB peaked in August at Havoc Meadow of 233.1 g/m^2^. The lowest SPAD measured Chlorophyll proxy of 16.06 was recorded in a quadrat at Havoc Meadow, whilst the highest of 47.2 was recorded in a quadrat at Lindean Moor. Mean quadrat SPAD measured chlorophyll proxy peaked in May at Murder Moss with a value of 37.2.

### Spectral-species diversity relationship

**Fig. 6:**
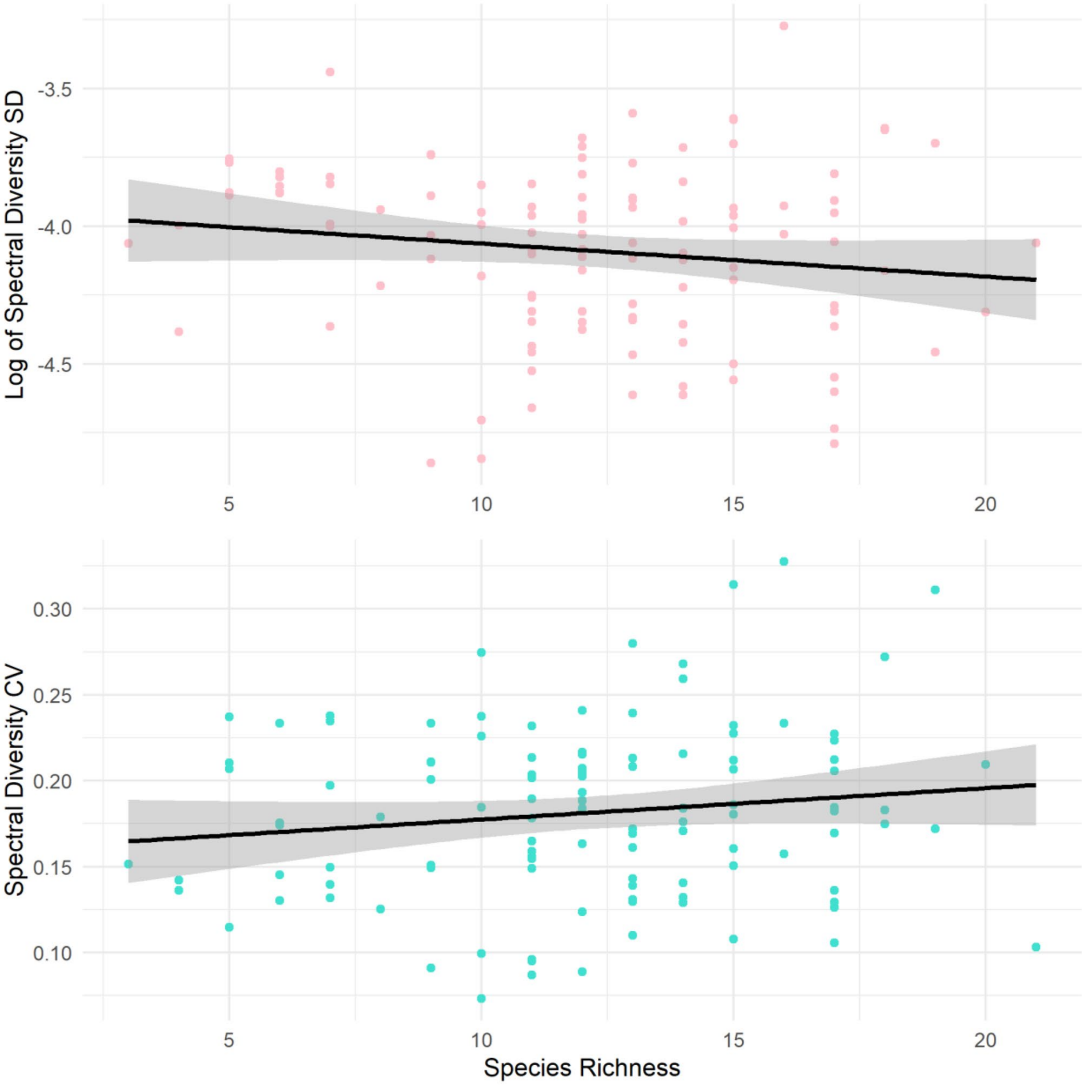
The relationship between species richness and spectral diversity: logarithmic of the standard deviation (pink) and coefficient of variation (blue) across seven species-rich grassland sites in Scotland. The black lines represent the fitted linear regression model with a 95% confidence interval (shaded areas)

There was no significant relationship between species diversity and the log of spectral diversity SD (*t* = −1.790, df = 112, *p* = 0.120, 95% CI [−0.32, 0.04]), and spectral diversity SD showed a low predictive power for species diversity (R^2^ = 0.0214). Similarly, the relationship between species richness and spectral diversity CV was not statistically significant (*t* = 1.482, df = 112, *p* = 0.141, 95% CI [−0.05, 0.31]), which demonstrated a comparably low predictive power (R^2^ = 0.0192) (Fig. 6).

### Species-rich grassland community trait retrieval

**Fig. 7:**
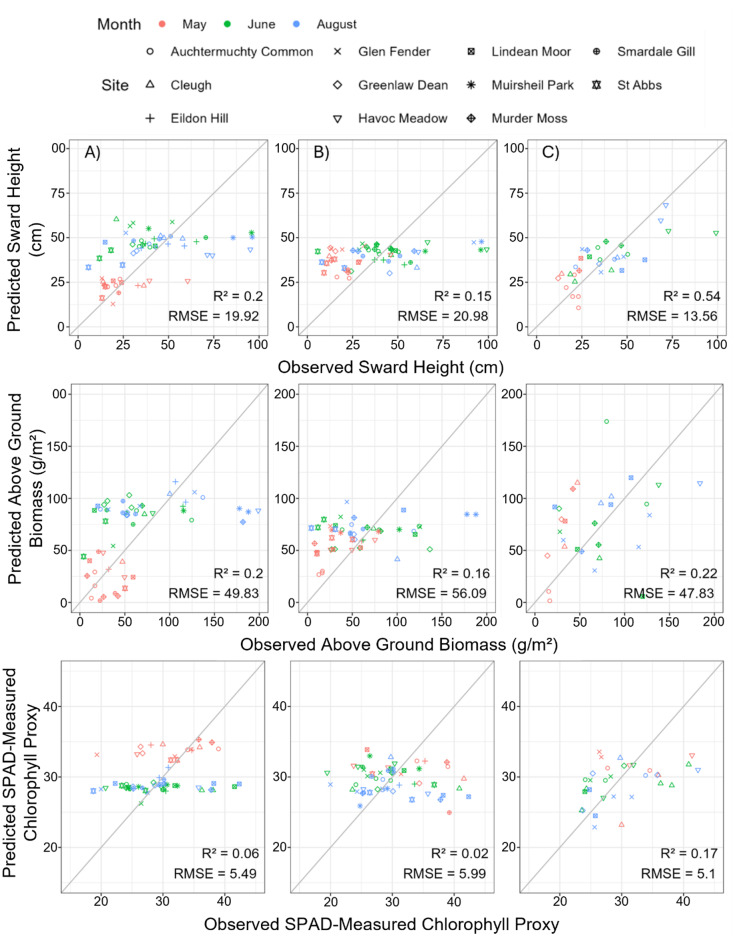
Predicted versus observed sward height (cm), above ground biomass (g/m^2^), and average Soil Plant Analysis Development (SPAD) used as a proxy for chlorophyll from (**A**) Sentinel-2 in 10 m resolution, (**B**) Planetscope in 3 m resolution, and (**C**) Micasense MX red-edge dual camera in 8 cm resolution models

The final PLS models showed mostly low trait predictive power across the RS sensors. The MS data had the strongest predictive power for all traits: sward height (R^2^ = 0.545, RMSE = 13.56 cm, removing the RGB and SWIR surface reflectance band values and spectral diversity metrics from the model), AGB (R^2^ = 0.221, RMSE = 48.26 g/m^2^, removing the RGB, NIR, and SWIR surface reflectance bands from the model and the red-edge VIs), and SPAD (R^2^ = 0.167, RMSE = 5.10, removing only the blue band value from the model). PS models consistently performed poorly for trait prediction (Fig. 7).

The final models for trait prediction are: PLS-MS for AGB, sward height, and SPAD (Table 2). The sensitivity of various combinations of predictor variables from the Micasense sensor can be visualised in Fig. S1 and are discussed in supplementary materials 1. Comparisons of trait retrieval with and without spectral diversity metrics can be found in Table S4.

**Table 2:** The combination of final inputted predictor variables in the final partial least square regression models (in bold) used for species-rich grassland community trait estimation with corresponding best sensor for prediction. Spectral band (B) numbers are listed in the models, with abbreviated vegetation indices, detailed in Table S3

Community Trait	Final Sensor for Model	Inputted Predictor Variables		
		Spectral Bands	Vegetation Indices	Spectral Diversity Metric
Sward Height (cm)	Micasense (PLS-MS)	NIR, Red-Edges	NDVI, GVI, EVI, NDVIRE1, NDVIRE2, NDVIRE3, NDRE1, NDRE2	None
		**sward_height ~ B4 + B5 + B9 + B10 + NDVI+ GVI+ EVI+ NDVIRE1 + NDVIRE2 + NDVIRE3 + NDRE1 + NDRE2**		
Above Ground Biomass (g/m^2^)	Micasense (PLS-MS)		NDVI, GVI, EVI, NDVIRE1, NDVIRE2, NDVIRE3, NDRE2	CV and SD
		**AGB ~ NDVI + GVI + EVI + NDVIRE1 + NDVIRE2 + NDVIRE3 + NDRE2 + ave_cv + ave_sd**		
SPAD-measured Chlorophyll-proxy	Micasense (PLS-MS)	Blue-444, Red-650, Green-531, Red-Edge 3, NIR, Green, Red	NDVI, GVI, EVI, NDVIRE1, NDVIRE2, NDVIRE3	CV and SD
		**SPAD ~ B2 + B3 + B4 + B6 + B7 + B8 + B10 + NDVI + GVI + EVI + NDVIRE1 + NDVIRE2 + NDVIRE3 + ave_cv + ave_sd**		

## Discussion

This study aimed to investigate the retrieval of species richness and other grassland community plant traits by RS to distinguish SRGs in Scotland. It is one of few extensive explorations of RS data, that incorporates multiple spectral and spatial resolutions specifically for trait retrieval, in a broad range of Scottish semi-natural SRGs. Through these objectives, we hoped to aid in the differentiation and monitoring of threatened habitats in Scotland using RS approaches, that could be applied more broadly to grassland conservation.

### Species – spectral diversity relationship

Our results showed that there was no significant relationship between species richness and spectral diversity across the seven study sites where this was investigated. Although there have been a range of studies [80] investigating the Spectral Variation Hypothesis, none have been so extensive across a range of semi-natural heterogenous grasslands, at such a fine spatial resolution as this.

Both spectral diversity metrics (CV and SD) were poor predictors of species richness, explaining approximately 2% of variation seen. Whilst this challenges the Spectral Variation Hypothesis, the results align with many similar studies indicating no clear relationship between spectral and species diversity in grasslands (e.g. [41, 80, 81]). Particularly, the case seems to be most controversial within grassland habitats, where the prediction performance of the Spectral Variation Hypothesis has seen greatest variability [41, 82, 83]. In fact, Conti et al. [84] found negative relationships between their spectral diversity and species diversity metrics, supporting the negative (although not significant) relationship between spectral diversity SD and species richness we observed. To date, studies that have demonstrated positive relationships between spectral and species diversity have been undertaken in more homogenous, species-poor ( < 10 species), or experimental grassland plots (e.g. [36, 38, 69]). Imran et al. [69] summarised these differences well, highlighting the reduced prediction power of spectral diversity metrics in a species-rich grassland versus a species-poor grassland.

Many confounding factors could result in the low confidences found in this relationship, including biomass, species abundances, sward structure, bare soil presence, and spatial resolution issues [43, 85]. However, when originally choosing modelling parameters to investigate the Spectral Variation Hypothesis, biomass, sward structure, and sward height variation (SD) were included in linear-mixed effect models and found to have little influence on variation ( < 5%). As such, these variables were not included as confounding factors in the final linear models investigating the relationship between species and spectral diversity. Furthermore, we noted that all quadrats had greater than 99% vegetation cover, thus, likely not influencing our results here.

Both spatial and spectral resolutions are known limitations in grassland RS [30, 86]. Indeed [69], reported increased spatial resolution resulted in a more positive relationship between spectral and species diversity across the visible region compared to a weaker relationship across the near infrared region. The spatial resolution of the sensor used in our study is 8 cm (within the range defined by [38] as suitable for the application of this analysis). Perhaps even finer spatial resolution would need to be evaluated for such diverse grasslands as those in our study.

Our spectral diversity metrics were averaged across 10 bands (multispectral) compared to studies such as that by Möckel et al. [87] where spectral diversity metrics were calculated across 245 bands (hyperspectral). As such, the reduced amount of surface reflectance (spectral diversity) information across wavelengths may influence our results. Although the Micasense sensor we used captures narrow wavebands, which typically outperform broad waveband sensors due to increased sensitivity to small changes in spectra [88, 89], the number of bands could still be limiting here.

**Fig. 8:**
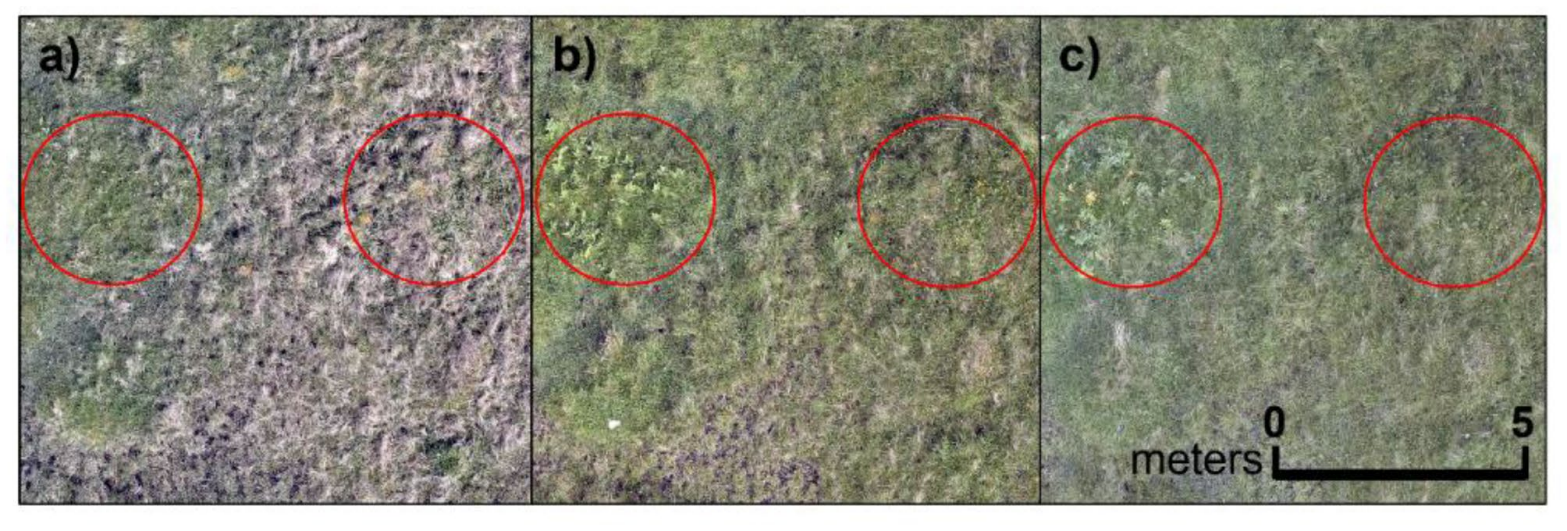
Phenological differences of vegetation across one site during the growing season **a**) May, the start of surveying, **b**) end of June, the peak of the survey season, and **c**) August, the end of surveying season, including new growth, increased flowering (circled in red), green-up, then dye-off (yellowing) of vegetation. Images captured with a zenmuse P1 camera flown at 50 m

Phenological stage is also key in grassland monitoring and may have influenced our results, as we measured across a season (Fig. 8). Thornley et al. [90] found that phenological diversity (defined as the number of phenological stages in a plot) confounded the Spectral Variation Hypothesis, suggesting that the timing of data collection be crucial in determining whether a relationship between spectral and species diversity can be seen. Species functional group (e.g., graminoids, forbs, legumes, and bryophytes) will also affect the spectral response of a grassland community, as will extreme weather events (drought/flooding) and diseases [91]. These are not variables we considered in our analysis and their inclusion in further research that explores the hypothesis is warranted.

We were not able to measure the abundance of each species in our quadrats; however, this is known to confound the species – spectral diversity relationship, as does the abundance of each plant functional group [86]. Indices that account for species abundance and evenness (Simpson’s diversity and Shannon’s diversity) have shown positive relationships with spectral diversity [30, 86]. Potentially, the impact of species and functional group abundance is high in heterogenous grasslands, therefore, the dominance of certain species may interfere with the interpretation of the species-spectral diversity relationship. We acknowledge that this may alter the relationship we see between spectral and species diversity we report, where spectral diversity is less able to capture solely species richness but instead other aspects of diversity. For instance, the hypothesis has better explained functional diversity in some grassland studies [81, 92].

### Grassland community trait estimation

We found low predictions (all but one R^2^ < 0.5) from the PLS models for trait estimation, inconsistent with recent studies [22, 93, 94]. AGB has often been well predicted by VIs, however, these studies typically consider homogenous, agricultural, or experimental grasslands to assess productivity or grazing intensity [93–96]. In more complex vegetation types such as SRGs, with diverse structural and functional traits, the retrieval accuracy of properties such as leaf area index and AGB is reduced (e.g. [97–99]). These difficulties could be further enhanced depending on the time of data collection, where presence of senescent material could significantly alter retrieval success [100]. It appears that the application of previously successful predictive models (R^2^ > 0.5) may not be suitable across various grassland types under differing biotic and abiotic conditions. This may explain the range in model performances seen across studies, including our own low R^2^ values, with large variation both within and across sites [101].

Additionally, most of our quadrats had a high proportion of flowering plants, previously seen to negatively affect the predictive power of statistical models in the retrieval of plant traits [102]. Zhang et al. [77] found that model prediction for structural properties, such as AGB and plant height was similarly low (explaining <45% variance) in natural heterogenous grasslands, however, they achieved high R^2^ values for chlorophyll content. Conversely, in our study, SPAD was most poorly predicted with all models explaining less than 20% of the variation. This could be explained by the number of heterogenous study sites whereby senescence may highly vary both within and across sites, or the methodology associated with measuring chlorophyll.

Spectrophotometers are known chlorophyll extractors in the lab. A SPAD meter measures the amount of light transmitted by certain wavelengths and is proportional to the amount of chlorophyll within a leaf [103]. As such, it is instead a proxy of chlorophyll content. However, Ludwig et al. [104] demonstrated that after calibration the relationship between SPAD values and actual chlorophyll content was not strong in a semi-natural grassland, and SPAD measurements were poor predictors of total leaf chlorophyll content (all models R^2^ = < 0.5). Therefore, the use of a SPAD meter for plant communities, rather than for specific species, in heterogenous grasslands may not be applicable, although further research may be required.

The predicted versus observed plots (Fig. 7) also showed that some sites do have extreme values in the structural traits and were far from the regression line, potentially reducing the models’ predictive power. These identified sites tended to have more dominant tall growing grasses e.g*., Deschampsia cespitosa*, and potentially this influenced the model results. We can see from Fig. 5 that many sites had large ranges in trait values, and we observed high intraspecific variation of traits within species measured in our quadrats. Siefert et al. [105] demonstrated that intraspecific variation can influence 25% of variation within community level traits. Further exploration of the result surrounding this concept is included in the Supplementary Materials 1, Table S5/Fig. S2.

Grasslands are varied by nature, showing quick responses to changes in conditions, climate, and management. As such, the success of trait prediction may vary between years, for example if a confounding factor has a greater influence on trait prediction which is reflected in the grassland community response, as explained by Thornley et al. [80], and that collecting data across seasons invites even further variation into the data. The temporal mismatch between in situ surveys and the satellite acquisitions may further impact this, as the timing of satellite acquisitions is important in RS applications [106, 107]. Further research, plus personal observation, suggests that other topographical variables are important in influencing grassland characteristics e.g., elevation, slope, and aspect [108]. It could be that predictive models for grassland community structural traits are best estimated with the inclusion of both spectral and topographical data. With this in mind, the difference in R^2^ values at a specific site per year may be useful for monitoring purposes and assessing annual change.

### Sensor comparison for trait retrieval

The Micasense performed the best out of the three sensors, suggesting that trait prediction is spatially limited in SRG RS. Capolupo et al. [74] note that the resolution should reflect the size of the desired trait to be estimated. For some traits, PLS-S2 models only performed slightly worse than the PLS-MS models, particularly for AGB (Fig. 7). The differences seen here in the R^2^ values between the sensor responses could be due to the variation seen on canopy versus individual level. Due to the pixel size of the MS camera, this is likely to be capturing species level data despite being averaged across the canopy. This could explain why the differences in the R^2^ values are much larger for SPAD and sward height – which, although were averaged across the canopy, the data were collected from individual plants – compared to AGB.

The results from the models may also suggest that spatial resolution is a greater limiting factor only when spectral resolution is not: PS consistently performed the worst, despite having a higher spatial resolution than S2 but has a lower spectral resolution. Depending on the application, S2 may outperform PS [109], as we see here. We argue this is most likely because of the inclusion of a greater number of red-edge narrowbands, as well as short-wave infrared bands which PS lacks, as seen in Zagajewski et al. [109]. Thus, a greater number of VIs could also be calculated for S2, providing further spectral information that would improve trait estimation to a larger extent than the greater spatial resolution of PS would provide.

### Limitations

Certain measurements were not considered in this study (e.g., species abundance) due, primarily, to resource (time and labour) requirements. We were unable to further explore the influences of other confounding factors (such as phenology and functional traits) on the relationship between spectral and species diversity due to these limitations. It is possible that the inclusion of these variables may help improve the ability of RS data to predict species richness and should be considered in future research.

Other spectral diversity metrics could be tested to see how these influence the results of the diversity estimates, for example clustering techniques such as the Spectral Species approach [71]. These were not tested here, as they are less widely applied to the Spectral Variation Hypothesis [80] and assumes that one pixel only captures one species. We did not think this suitable for our analysis where one pixel, even at 8 cm, likely includes the presence of multiple species [110]. However, if able to bypass the issues of cross-site comparisons, this could be more appropriate for the handling of outliers that would be seen in these heterogenous grasslands.

Additionally, Thornley et al. [80] identify that, at canopy level, grasslands are prone to quick responses to environmental and management change. Our study sites did consist of a range of management regimes which may have impacted the results. If possible, these considerations should be measured and included in the methods and analysis steps in the future.

Moreover, due site restrictions and access permissions, UAV data could only be collected at seven sites. This means we were unable to test the Spectral Variation Hypothesis in all SRG classes. Perhaps the Spectral Variation Hypothesis is more successful in specific classes of SRGs and requires further research.

Scotland is also a country with highly changeable weather conditions; from May to August 2022 temperatures ranged from averages of 6.9–18.1 °C, whilst total rainfall varied from 80.6–121 mm [111]. Consequently, retrieval of satellite imagery (also limited by revisitation times) on the same day as in situ sampling was not always possible. Images that were cloud free over the site quadrats were acquired as close as possible to the field dates. We originally tested retrieval abilities from satellite information by lowering the threshold between the survey day and the satellite image, acquisition but we found this made little improvement to our results. However, where possible, we express this must be as close to the field survey day where feasible. Likewise, poor weather conditions meant that the MS could not fly for some field dates, restricting our data collection.

Finally, we note that the very low trait retrieval of SPAD-measured chlorophyll is likely primarily due to the methodological limitations of using a SPAD meter for estimating chlorophyll in diverse grasslands. Time and equipment limitations meant we were not able to conduct lab extraction of leaf chlorophyll content and we recommend that further analysis be conducted with lab-measured chlorophyll to see how this may improve retrieval accuracy of this trait, specifically for SRGs.

### Future recommendations

Time, labour, and financial costs will restrict the amount of data that can be further collected to enhance RS of SRGs. Ideally, future research can incorporate the collection of species abundance, species functional group, number of flowering plants, and plant phenological stage to be considered as fixed effects [86, 90, 91, 102]. Data collection should make note of management practices, presence of disease, and weather conditions, specifically any unusual events as random effects [80]. Temporal variation and impact of senescence can be reduced by collecting data during one phenological stage, aligning any mapping attempts to this stage [90]. Where possible, influencing environmental determinants e.g., topographical variables should be included [108], whilst the sensor and spatial resolution should be considered per trait estimation. For example, the use of active sensors such as Sentinel-1 (most commonly used for biomass estimation in grasslands [112]) can be explored, especially in combination with optical sensors, as seen in Bartold et al. [113]. Further investigations can also assess the integration of spectral diversity metrics in grassland community trait retrieval as some improvements in predictions were shown with this inclusion in this study (Table S4).

Our initial exploration highlights the difficulties in using typical RS approaches in diverse grasslands. This study is the first of its kind that uses common RS applications, utilising multiple sensors across SRG classes, and emphasises that these systems may require further tailoring of methods to accurately capture the diversity and traits of these habitats. The use of ground surveys that measure the fixed and random effects we mention, across multiple grassland types, may improve the model predictions. To do so, interdisciplinary research and collaboration between ecologists and remote sensing scientists should be prioritised in project designs. This can be facilitated with the use of collaborative platforms and readily available data (such as from citizen science) to increase data collection capacity and knowledge integration. As such, we encourage the development of context-specific RS workflows that can be investigated to distinguish between grasslands of high conservation value for their protection.

## Conclusions

SRGs are a priority habitat in Scotland and require increased directed monitoring. While RS applications have the potential to broaden the spatial scale of SRG assessment, to date, this has not been widely tested. By exploring RS applications in 7–11 SRGs in Scotland, we investigated the Spectral Variation Hypothesis, modelling the relationship between species and spectral diversity, and explored trait retrieval of grassland community AGB, sward height, and a SPAD measured chlorophyll proxy using predictive modelling. These common methods were integrated by utilising spectral diversity as a predictor variable in trait retrieval, an area that has had little exploration.

The results from this study highlight potential barriers to the use of prediction estimates from RS in distinguishing and monitoring semi-natural SRGs. Nevertheless, while most of the R^2^ values are low, some variation is explained by RS data, especially for structural trait estimation, that holds potentially important information (particularly in relation to the selection of predictor variables). The many considerations from these diverse and dynamic grassland habitats make it difficult to apply previous theories and common modelling approaches to SRGs. It appears that spectral variation in SRGs is not majorly linked to species counts but may more likely be representative of the species functional group, dominance, and environmental variance that is experienced regularly in these habitats.

This work does not expressly discount earlier findings or theories surrounding the retrieval of grassland parameters from RS data but now adds to the body of literature that express difficulties in these diverse habitats, especially in relating species and spectral diversity. It is apparent that RS data alone may not yet be enough to predict certain plant community traits in SRGs with high accuracy. We highlight that RS of SRGs is entirely community and site context dependent which may hinder progress in this area and encourage context specific design in remote sensing applications that may be more suitable when monitoring across multiple natural and semi-natural grassland classes.

## Electronic supplementary material

Below is the link to the electronic supplementary material.

Supplementary material 1

Supplementary material 2

## Data Availability

The datasets generated during and/or analysed during the current study are available in the supplementary materials 2.
