# Supplementary material for: Predicting species diversity and community traits from remote sensing in species-rich grasslands
Source: BMC Ecol Evol. 2026 Feb 13;26:21. doi: 10.1186/s12862-026-02500-4 (PMC12937569; doi:10.1186/s12862-026-02500-4)
Supplement: Supplementary file 1 — Supplementary material 1 [file 12862_2026_2500_MOESM1_ESM.docx]

## Supplementary Materials 1

Table S1. Study site names with their corresponding codes.

| **Site Name** | **Site Code** | **Coordinates (Latitude, Longitude)** | **Elevation (m)** |
| --- | --- | --- | --- |
| Auchtermuchty Common | AC | 56.304206 , -3.2298358 | 115 |
| Cleugh | CL | 55.155136 , -4.1783171 | 155 |
| Eildon Hill | EH | 55.587711, -2.699032 | 240 |
| Greenlaw Dean | GD | 55.723180 , -2.4892521 | 198 |
| Glen Fender | GF | 56.788592 , -3.8104338 | 333 |
| Havoc Meadow | HM | 55.943391 , -4.5939319 | 4 |
| Lindean Moor | LM | 55.550283 , -2.7881956 | 248 |
| Murder Moss | MM | 55.547678 , -2.7860838 | 246 |
| Muirsheil Park | MP | 55.831489 , -4.6982657 | 224 |
| St Abb’s | SA | 55.911320 , -2.1407472 | 60 |
| Smardale Gill | SM | 54.453803 , -2.4264479 | 239 |

**Table S2. Comparison of band information across three sensors: Sentinel-2A (S2), Planetscope (PS), and Micasense MX Red-edge 5.5 dual camera (MS).**

| **Band** | **Band Number** | | | **Central Wavelength (nm)** | | | **Band Width (nm)** | | |
| --- | --- | --- | --- | --- | --- | --- | --- | --- | --- |
|  | *S2* | *PS* | *MS* | *S2* | *PS* | *MS* | *S2* | *PS* | *MS* |
| Coastal Blue | 1 | 1 | 6 | 443 | 443 | 444 | 21 | 20 | 28 |
| Blue | 2 | 2 | 1 | 490 | 490 | 475 | 66 | 50 | 32 |
| Green-531 |  | 3 | 7 |  | 531 | 531 |  | 36 | 14 |
| Green | 3 | 4 | 2 | 560 | 565 | 560 | 36 | 36 | 27 |
| Yellow |  | 5 |  |  | 610 |  |  | 20 |  |
| Red-650 |  |  | 8 |  |  | 650 |  |  | 16 |
| Red | 4 | 6 | 3 | 665 | 665 | 668 | 31 | 31 | 14 |
| Vegetation Red-Edge | 5 | 7 | 9 | 705 | 705 | 705 | 15 | 15 | 10 |
| Vegetation Red-Edge |  |  | 5 |  |  | 717 |  |  | 12 |
| Vegetation Red-Edge | 6 |  | 10 | 740 |  | 740 | 15 |  | 18 |
| Vegetation Red-Edge | 7 |  |  | 783 |  |  | 20 |  |  |
| NIR | 8 |  | 4 | 842 |  | 842 | 106 |  | 57 |
| Narrow NIR | 8A | 8 |  | 865 | 865 |  | 21 | 40 |  |
| SWIR | 11 |  |  | 1610 |  |  | 91 |  |  |
| SWIR | 12 |  |  | 2190 |  |  | 175 |  |  |

**Table S3. The vegetation indices calculated per sensor: Normalised Difference Vegetation Index (NDVI), Enhanced Vegetation Index (EVI), Greenness Vegetation Index (GVI), Sentinel-2 Red-Edge Position Index (S2REP), Normalised Difference (ND) Red-Edge indices (NDVI red-edge/ND red-edge), and the Normalised Difference Infrared Index (NDII)~~.~~ Band numbers are listed by central wavelength (nm).**

| **Vegetation Indices** | **Sentinel-2A** | **PlanetScope** | **Micasense MX Red-Edge Dual camera** | **Source** |
| --- | --- | --- | --- | --- |
| NDVI | (B8_(842)_-B4_(665)_)/(B8_(842)_+B4_(665)_) | (B8_(865)_-B6_(665)_)/(B8_(865)_+B6_(665)_) | (B4_(842)_-B3_(668)_)/(B4_(842)_+B3_(668)_) | Imran *et al*., 2020; Peciña et al., 2021; Qin *et al.,* 2021 |
| EVI | 2.5(B8_(842)_-B4_(665)_)/(B8_(842)_+6 B4_(665)_-7B2_(490)_+1) | 2.5(B8_(865)_-B6_(665)_)/(B8_(865)_+6 B6_(665)_-7B2_(490)_+1) | 2.5(B4_(842)_-B3_(668)_)/(B4_(842)_+6B3_(668)_-7B1_(475)_+1) | Peciña et al., 2021; Qin *et al.,* 2021; Zou *et al.,* 2022 |
| GVI | (B8_(842)_-B3_(560)_)/(B8_(842)_+B3_(560)_) | (B8_(865)_-B4_(565)_)/(B8_(865)_+B4_(565)_) | (B4_(842)_-B2_(560)_)/(B4_(842)_+B2_(560)_) | Peciña et al., 2021 |
| S2REP | 705+35((2(B7_(783)_+B4_(665)_)-(B5_(705)_)/(B6_(740)_–B5_(705)_)) |  |  | Li *et al.,* 2021; Zou *et al.,* 2022 |
| NDVI_Red-Edge 1_ | (B8_(842)_-B5_(705)_)/(B8_(842)_+B5_(705)_) | (B8_(865)_-B7_(705)_)/(B8_(865)_+B7_(705)_) | (B4_(842)_-B9_(705)_)/(B4_(842)_+B9_(705)_) | Li *et al.,* 2021; Peciña et al., 2021 |
| NDVI_Red-Edge 2_ | (B8_(842)_-B6_(740)_)/(B8_(842)_+B6_(740)_) |  | (B4_(842)_-B10_(740)_)/(B4_(842)_+B10_(740)_) | Li *et al.,* 2021; Peciña et al., 2021 |
| NDVI_Red-Edge 3_ | (B8_(842)_-B7_(783)_)/(B8_(842)_+B7_(783)_) |  | (B4_(842)_-B5_(717)_)/(B4_(842)_+B5_(717)_) | Li *et al.,* 2021; Peciña et al., 2021 |
| ND_Red-Edge1_ | (B6_(740)_–B5_(705)_)/( B6_(740)_+B5_(705)_) |  | (B10_(740)_–B9_(705)_)/( B10_(740)_+B9_(705)_) | Li *et al.,* 2021; Imran *et al*., 2020 |
| ND_Red-Edge2_ | (B7_(783)_–B5_(705)_)/( B7_(783)_+B5_(705)_) |  | (B5_(717)_–B9_(705)_)/( B5_(717)_+B9_(705)_) | Li *et al.,* 2021; Imran *et al*., 2020 |
| NDII | (B8_(842)_–B11_(1610)_)/( B8_(842)_+B11_(1610)_) |  |  | Li *et al.,* 2021; Qin *et al.,* 2021 |


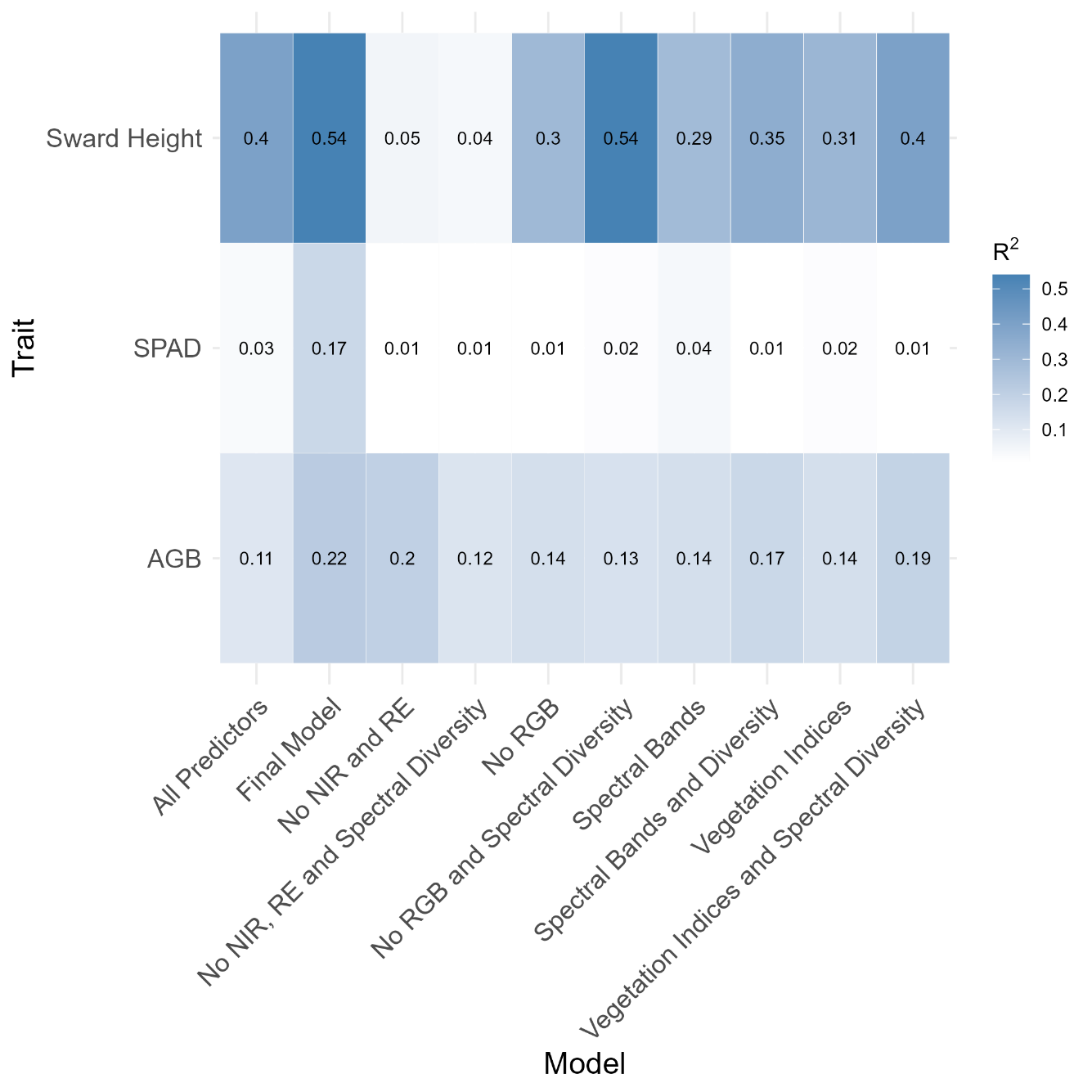


Figure S1. Variation in partial least square model performance on species-rich grassland trait prediction (sward height, SPAD measured chlorophyll proxy, and above ground biomass) using multiple combinations of predictor variables, testing the removal of vegetation indices, spectral bands including near infra-red (NIR), red-edge (RE), red green blue (RGB), and spectral diversity metrics.

We see that different regions of the electromagnetic spectrum respond differently to plant features, thus model performance for grassland trait retrieval subsequently changes when various combinations of predictor variables are used. For example, we see a decrease in retrieval ability of grassland sward height when we remove the NIR bands from the model (Fig. S1), likely because the NIR region is known to interact with the internal structural components of plants and is, therefore, useful in differentiating longer versus shorter sward heights because of the differing internal scattering of the NIR wavelengths^[[1]](#footnote-1)^.

The model success for AGB retrieval, however, is less broad with the multiple model iterations. The use of vegetation indices (VIs) provides more accurate trait retrieval than with a combination of the single reflectance bands, however. VIs can be more sensitive to AGB than the raw reflectance band signals alone due to noise reduction^[[2]](#footnote-2)^. The NIR region did not interact the same with AGB as with sward height, which may be due to the co-variation between structural and biochemical traits^1^. Although we know that VIs have potential to retrieve AGB well, strong retrieval accuracy may not have occurred here due to AGB saturation^[[3]](#footnote-3)^ in our SRG sites, which may have greater biomass due to infrequent mowing or grazing. The issues surrounding greater retrieval accuracy are further discussed in the main manuscript.

The model iterations did very little to improve trait retrieval for SPAD-measured chlorophyll, and we note this is likely a methodological limitation with the use of a SPAD-meter in diverse grasslands, discussed in detail in the main manuscript. Information on chlorophyll retrieval is both seen in the absorption in the visible region and reflectance in the NIR region. As such, bands or VIs that capture these regions of the electromagnetic spectrum are beneficial. It is likely that responses from the spectrum across the regions were needed for this trait retrieval and the use of chlorophyll sensitive VIs enhanced this, but not to a great extent^[[4]](#footnote-4)^. Further exploration of predictor sensitivity to chlorophyll content would be needed combined with lab measurements of leaf chlorophyll.

Further trait information may be found within spectral diversity that is currently not known. It was not possible to use spectral diversity metrics for S2 and PS data per quadrat for trait estimation. Spectral diversity metrics could have been determined for S2 and PS data by averaging quadrat surface reflectance values across each site (to give one SD or CV value per site for both S2 and PS). However, this would result in only eleven data points per sensor when setting up the PLS models, which was not suitable for train/test data partitioning.

The addition of the MS spectral diversity metrics into the PLS-MS models did slightly improve prediction estimates for AGB, fresh weight, and SPAD but reduced the prediction power of the model for sward height (Table S4).

Table S4. Partial least square regression model results for grassland trait estimation with and without the inclusion of spectral diversity metrics derived from a Micasense MX red-edge dual camera. Improved prediction estimates are highlighted in bold.

| **Trait** | **With spectral diversity metrics** | | **Without spectral diversity metrics** | |
| --- | --- | --- | --- | --- |
|  | *R^2^* | *RMSE* | *R^2^* | *RMSE* |
| Sward Height (cm) | 0.305 | 16.54 | **0.545** | **13.56** |
| Above Ground Biomass (g/m^2^) | **0.221** | **48.26** | 0.130 | 47.74 |
| Fresh Weight (g) | **0.235** | **98.74** | 0.209 | 99.07 |
| SPAD (Chlorophyll-proxy) | **0.167** | **5.10** | 0.160 | 5.13 |

The results were further explored to investigate whether specific sites had a greater influence on low R^2^ values for trait prediction and why that might be, with the previous data collected over 2021. The data showed little consistency in the variation between sites or traits (Table S5). There was some suggestive evidence that increasing variation in a trait (i.e., greater range in trait values) resulted in lower predictive power of the PLS models. This relationship was tested with Pearson’s Correlation coefficients for all our investigated traits, resulting in negative trends. However, this was only significant for sward height (p = 0.0368).

Table S5. Investigation of predictive power of Sentinel-2 data across the 11 study sites, plus an additional five sites surveyed in 2021, across 2021, 2022, and both years combined.

| **Site** | **Year** | ***n*** | **S2** | | | | | |
| --- | --- | --- | --- | --- | --- | --- | --- | --- |
|  |  |  | **AGB (g/m^2^)** | | **Sward Height (cm)** | | **SPAD-measured chlorophyll-proxy** | |
|  |  |  | *R^2^* | *RMSE* | *R^2^* | *RMSE* | *R^2^* | *RMSE* |
| AC | 2021 | 18 | 0.289 | 27.35 | 0.299 | 15.77 |  |  |
| AC | 2022 | 18 | 0.282 | 38.67 | 0.406 | 12.62 | 0.185 | 10.52 |
| AC | both | 36 | 0.117 | 41.91 | 0.503 | 9.07 |  |  |
| BL | 2021 | 18 | 0.655 | 18.72 | 0.706 | 8.00 |  |  |
| CL | 2021 | 18 | 0.832 | 14.92 | 0.823 | 12.19 |  |  |
| CL | 2022 | 18 | 0.929 | 23.37 | 0.924 | 6.65 | 0.027 | 6.62 |
| CL | both | 36 | 0.318 | 37.93 | 0.741 | 8.38 |  |  |
| EH | 2021 | 18 | 0.490 | 48.00 | 0.586 | 26.67 |  |  |
| EH | 2022 | 18 | 0.925 | 28.49 | 0.622 | 14.97 | 0.655 | 5.98 |
| EH | both | 36 | 0.168 | 37.94 | 0.051 | 21.83 |  |  |
| GF | 2021 | 18 | 0.094 | 130.18 | 0.290 | 34.83 |  |  |
| GF | 2022 | 18 | 0.284 | 34.02 | 0.022 | 27.89 | 0.789 | 31.06 |
| GF | both | 36 | 0.156 | 41.03 | 0.013 | 39.80 |  |  |
| GL | 2021 | 18 | 0.137 | 49.81 | 0.568 | 13.82 |  |  |
| GD | 2021 | 17 | 0.402 | 37.73 | 0.879 | 7.62 |  |  |
| GD | 2022 | 18 | 0.0001 | 36.67 | 0.399 | 12.99 | 0.242 | 3.14 |
| GD | both | 35 | 0.347 | 36.32 | 0.390 | 14.30 |  |  |
| GH | 2021 | 18 | 0.429 | 25.03 | 0.380 | 20.13 |  |  |
| HM | 2021 | 23 | 0.669 | 45.61 | 0.261 | 18.61 |  |  |
| HM | 2022 | 18 | 0.226 | 140.08 | 0.883 | 9.24 | 0.922 | 7.60 |
| HM | both | 41 | 0.057 | 77.24 | 0.205 | 26.14 |  |  |
| HP | 2021 | 18 | 0.549 | 42.49 | 0.280 | 21.42 |  |  |
| LM | 2021 | 18 | 0.938 | 19.38 | 0.357 | 9.35 |  |  |
| LM | 2022 | 18 | 0.856 | 16.19 | 0.776 | 11.80 | 0.304 | 7.01 |
| LM | both | 36 | 0.118 | 32.92 | 0.878 | 10.11 |  |  |
| MP | 2021 | 18 | 0.219 | 30.82 | 0.110 | 28.22 |  |  |
| MP | 2022 | 15 | 0.676 | 32.56 | 0.901 | 19.34 |  |  |
| MP | both | 33 | 0.313 | 34.98 | 0.224 | 23.15 |  |  |
| MM | 2021 | 17 | 0.606 | 30.32 | 0.396 | 24.05 |  |  |
| MM | 2022 | 18 | 0.745 | 69.50 | 0.863 | 12.45 | 0.699 | 7.06 |
| MM | both | 35 | 0.500 | 31.77 | 0.465 | 11.31 |  |  |
| RP | 2021 | 18 | 0.682 | 40.45 | 0.922 | 10.96 |  |  |
| SM | 2021 | 17 | 0.326 | 19.83 | 0.121 | 21.36 |  |  |
| SM | 2022 | 18 | 0.962 | 17.36 | 0.195 | 21.02 | 0.441 | 5.13 |
| SM | both | 35 | 0.581 | 16.00 | 0.693 | 8.66 |  |  |
| SA | 2021 | 18 | 0.005 | 26.59 | 0.886 | 3.93 |  |  |
| SA | 2022 | 18 | 0.448 | 18.41 | 0.848 | 4.82 | 0.710 | 5.03 |
| SA | both | 36 | 0.111 | 23.58 | 0.102 | 6.59 |  |  |

The data also indicated that even within site variation may result in poor trait prediction, with certain sites having higher R^2^ values one year, with low values the next. For example, the PLS-S2 model predicted AGB well for Eildon Hill in 2022 (R^2^ = 0.925) but predicted AGB poorly in 2021 (R^2^ = 0.490). When combining data from both years, the model predictive power was reduced further (R^2^ = 0.168) (Fig. S1).


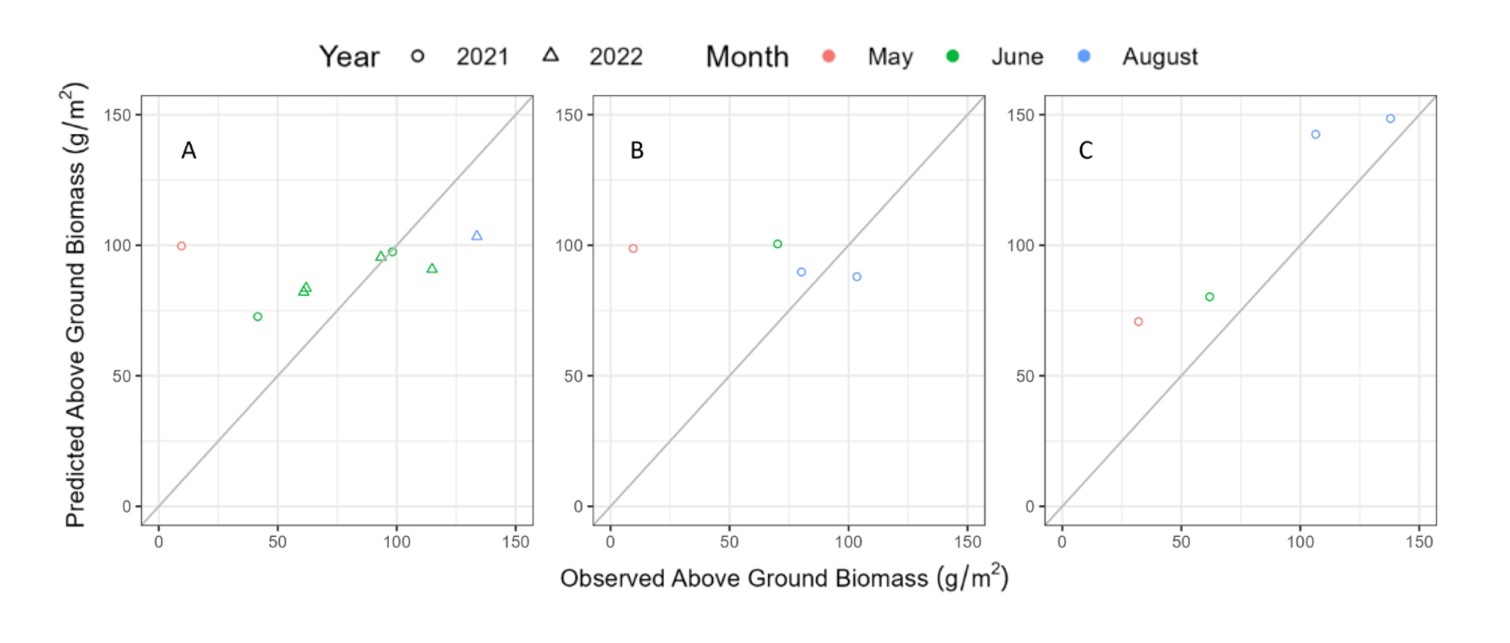


Figure S2. Predicted versus observed above ground biomass (g/m^2^) at Eildon Hill from Sentinel-2 in 10 m resolution surface reflectance values and vegetation indices from a) 2021 and 2022 combined, b) 2021, and c) 2022.

Supplementary Materials 2 (excel file of raw data used in analyses).

1. Imran HA, Gianelle D, Rocchini D, Dalponte M, Martín MP, Sakowska K, Wohlfahrt G, Vescovo L. VIS-NIR, red-edge and NIR-shoulder based normalized vegetation indices response to co-varying leaf and Canopy structural traits in heterogeneous grasslands. Remote Sensing. 2020 Jul 14;12(14):2254. [↑](#footnote-ref-1)
2. Meng B, Zhang Y, Yang Z, Lv Y, Chen J, Li M, Sun Y, Zhang H, Yu H, Zhang J, Lian J. Mapping grassland classes using unmanned aerial vehicle and MODIS NDVI data for temperate grassland in inner Mongolia, China. Remote Sensing. 2022 Apr 27;14(9):2094. [↑](#footnote-ref-2)
3. Wang G, Liu S, Liu T, Fu Z, Yu J, Xue B. Modelling above-ground biomass based on vegetation indexes: a modified approach for biomass estimation in semi-arid grasslands. International Journal of Remote Sensing. 2019 May 19;40(10):3835-54. [↑](#footnote-ref-3)
4. Bekkema, M.E. and Eleveld, M., 2018. Mapping grassland management intensity using sentinel-2 satellite data. GI_Forum, 2018(1), pp.194-213. [↑](#footnote-ref-4)
